# Actions following adverse drug events – how do these influence uptake and utilisation of newer and/or similar medications?

**DOI:** 10.1186/s12913-015-1165-9

**Published:** 2015-11-06

**Authors:** Nadia Barozzi, GMEE Geeske Peeters, Susan E. Tett

**Affiliations:** School of Pharmacy, The University of Queensland, Brisbane, QLD 4072 Australia; Healthy 4Life, PO Box 41, 4009 Basel, Switzerland; Schools of Population Health and Human Movement Studies, The University of Queensland, Brisbane, QLD 4072 Australia

**Keywords:** Pharmacoepidemiology, Rofecoxib, Bisphosphonates, Adverse drug events, Post-marketing surveillance

## Abstract

**Background:**

Over the last decade, actions following some adverse drug events received major publicity. This study investigated changes in usage patterns of medications in Australia following two examples - rofecoxib market withdrawal (2004) and warnings about jaw necrosis following bisphosphonates (2007).

**Methods:**

Dispensing data for COX-2 inhibitors (2000–2008) and anti-osteoporosis medications (2003–2012) were obtained from the Australian Pharmaceutical Benefits Scheme database. For bisphosphonates, data on Australian marketing expenditures were purchased from Cegedim^R^.

**Results:**

For COX-2 inhibitors, celecoxib dispensing halved after rofecoxib withdrawal, but meloxicam dispensing increased by 60 %. When lumiracoxib was introduced (2006) there was uptake of prescribing at a faster rate than meloxicam in 2002, its first year of use. For bisphosphonates, alendronate had highest use at the time of the warnings (8.3 DDD/1000/day), dropping to 4.9 DDD/1000/day by 2012. In contrast, risedronate use rose 2007–2012 from 4.1 to 4.9 DDD/1000/day. There was 49 % increase in reported annual expenditure on detailing for risedronate from 2007 to 2008 (to AUD$7.3 million) and only 29 % increase for alendronate (to AUD$3.1 million).

**Conclusions:**

The rapid uptake of prescribing of lumiracoxib and increased use of meloxicam flagged a concern, especially after rofecoxib withdrawal due to safety issues. Bisphosphonates are useful drugs, however the dramatic rise in expenditure on detailing, followed by a rise in utilisation of risedronate could suggest that adverse publicity triggered a marketing response. These examples highlight the importance of tracking utilisation of medication classes in real time, using different data as needed, to ensure that due caution is exercised (and quick intervention provided if needed) for medications in the same class.

**Electronic supplementary material:**

The online version of this article (doi:10.1186/s12913-015-1165-9) contains supplementary material, which is available to authorized users.

## Background

Post-marketing surveillance for prescribed medications has been receiving increasing attention, from academic researchers, prescribers, consumers and regulatory agencies [1–3]. The potential adverse influence of the pharmaceutical industry has also been identified amid calls for a more objective mission for medicine [4]. Rare adverse effects may not be appropriately captured during early clinical trials, which often recruit relatively well patients with few other conditions or illnesses and in insufficient numbers to detect rare events [5]. Deleterious effects may come to light when prescribing commences in the more general population [1, 5]. Special vulnerability may be due to age (both extremes of life, the elderly and paediatric populations), concurrent illnesses and polypharmacy. In ‘real life’, medications are used in combinations, not all of which can be tested in prior clinical trials, and in people with multiple conditions, treated and untreated [3, 5]. All too often adverse events caused by medications are found to be important only when effects become reported in populations after widespread prescribing [3]. If the use of the medication is already far reaching, perhaps prescribed to millions of people, there can be large absolute numbers of people affected by ‘rare’ side effects before action is taken, such as warnings issued by regulatory bodies or medications withdrawn from the market [1, 3].

Two recent examples of clinical adverse effects which caused significant safety problems in large numbers of individuals, were osteonecrosis of the jaw with bisphosphonates and increased risk of myocardial infarction following rofecoxib use. These adverse events and the effects on patient safety were the subject of mass media current affairs television programs in Australia [6, 7]. Warnings and safety alerts for bisphosphonate-related osteonecrosis of the jaw from regulatory agencies were issued 2005–2007 and received extensive public media coverage [7–10]. Alendronate was the most prescribed bisphosphonate in Australia at this time, and by implication was associated with this adverse event [11]. Evidence of rofecoxib-related increased risk of myocardial infarction led to its market withdrawal in September 2004 [3, 12–15]. Our premise was that widespread publicity about these adverse effects, in the public media and in professional publications and journals, would influence utilisation of other medications in the same class as the implicated drug(s), and especially influence more cautious prescribing behaviour for medications introduced after these adverse events in the same drug class(es).

There are, however, many influences on prescribing behaviour, in addition to publicity and regulatory actions or warnings about adverse events [4, 13]. Some of these include influences of the pharmaceutical industry, the effects of peer guidelines, perceived pressure from consumers or requests to prescribe, and new evidence from published clinical trials [4, 16, 17]. Patient preferences may also influence medicine choice, especially after media publicity or warnings of potential medication-related harms. Prescribing influences have been reviewed elsewhere [4, 18]. Thus new medications, or medicines perceived as somehow different from others in the same class which have been implicated in adverse effects, may perhaps be prescribed without due caution. At present Australia does not have a systematic way of monitoring use across medication classes, nor evaluating in real time the influence of adverse events reported for one medicine on prescribing of other medications.

The aim of this study was to investigate changes in usage patterns of key medications in Australia following rofecoxib market withdrawal (COX-2 inhibitor usage 2000–2008) and after the widespread warnings about bisphosphonates (usage 2003–2012), and the resulting publicity (both scholarly publications and public media) about these adverse medication events.

## Methods

Key dates for the two examples of adverse drug events causing large publicity and awareness were the market withdrawal of a COX-2 inhibitor, rofecoxib, in September 2004, and jaw necrosis from bisphosphonate use (in 2005 FDA warning added in USA; in December 2007 safety alert issued from Therapeutic Goods Administration in Australia), reports of which attracted extensive public media coverage and reports in health professional literature across Australia. Timelines for key events in the lifecycles of COX-2 inhibitors and bisphosphonates (subsidised as anti-osteoporosis medications) are shown in Tables 1 and 2.

**Table 1:** Timeline of regulatory and public subsidy decisions for COX-2 inhibitors in Australia

1999	*Jun*	Regulatory authority approved celecoxib for marketing in Australia
	*Oct*	Regulatory authority approved rofecoxib for marketing in Australia
2000	*Jun*	Rofecoxib recommended to be subsidized on the PBS as a treatment for osteoarthritis only
	*Aug*	Celecoxib listed on the PBS
2001	*Feb*	Rofecoxib listed on the PBS
	*Sep*	Regulatory authority approved meloxicam for marketing in Australia
2002	*Feb*	Meloxicam listed on the PBS
2004	*Jul*	Regulatory authority approved lumiracoxib for marketing in Australia
	*Sept*	Rofecoxib withdrawn from the Australian market
2006	*Aug*	Lumiracoxib listed on the PBS
2007	*Aug*	Lumiracoxib withdrawn from the Australian market

**Table 2:** Timeline of relevant activities potentially influencing utilisation of bisphosphonates in Australia (for further detail, see Peeters et al. [10])

2001	*PBS*	Alendronate, risendronate, etidronate available for post-menopausal women with established osteoporosis with minimal trauma fracture
2004	*ADRAC*	Reports of eye-problems related to bisphosphonates
2006	*ADRAC*	Reports of osteonecrosis of the jaw related to bisphosphonates
2007	*PBS*	Bisphosphonates, also includes women > 70years with BMD T score < −3.0
	*ADRAC*	Reports of renal impairment related to bisphosphonates, particularly zolendronic acid. Further reports of osteonecrosis of the jaw related to bisphosphonates
	*Media*	ABC television - The 7.30 Report on osteonecrosis of the jaw related to alendronate
	*TGA*	Safety Alert -advice issued December 2007 about bisphosphonate drugs and osteonecrosis of the jaw
2008	*PBS*	Zoledronic acid for patients with vertebral fracture
2009	*PBS*	Zolendronic acid, also includes women > 70years with BMD T score < −3.0

Dispensing data for COX-2 inhibitors (2000–2008) and bisphosphonates (2003–2012) for Australia were obtained from the publicly available national Pharmaceutical Benefits Scheme (PBS) administrative database (http://www.humanservices.gov.au/corporate/statistical-information-and-data/pharmaceutical-benefits-schedule-statistics/). The PBS provides subsidised pharmaceuticals to all Australian citizens and residents (complete national coverage). There are two levels of coverage, with General Beneficiaries (most of the population) paying a higher copayment (currently 37.70 Australian Dollars) than that paid by Concession Beneficiaries (currently 6.10 Australian Dollars for seniors and those receiving social security benefits). Data were downloaded for all PBS (including the veterans (Repatriation) scheme, RPBS) services for bisphosphonates subsidised for osteoporosis and for concession PBS services for COX-2 inhibitors (PBS item numbers shown in Additional files 1 and 2). PBS item numbers for bisphosphonates are indication-specific and those used in this study were only for osteoporosis (not for Paget’s Disease or other indications). Concession PBS services were chosen for COX-2 inhibitors as at this time dispensing for items below copayment was not captured and some of the COX-2 inhibitors fell below General copayment for some of that time period. The total number of services for each individual item number (one month supply; number of tablets/capsules specified by the recorded item number (Additional files 1 and 2)) was obtained for each relevant calendar year. Population numbers for Australia were obtained from the Australian Bureau of Statistics, and for the Concession population (seniors and social security beneficiaries) by request from Centrelink, a national government agency. Utilisation data were calculated as World Health Organisation (WHO) Anatomic Therapeutic Chemical (ATC) Defined Daily Doses (DDD) (2013) per 1000 head of population per day (DDD/1000/day = ((number of services * mg in each service)/DDD)/(population/1000)/365). Marketing expenditures for anti-osteoporosis medications (2003–2011) for Australia were purchased from Cegedim^R^ (www.cegedimstrategicdata.com), separated into the marketing ‘channels’. These marketing data have been described in more detail and used previously [19].

All data obtained were aggregated, with no identification possible. Ethics approval was not required for these publicly available data.

## Results

**Fig. 1:**
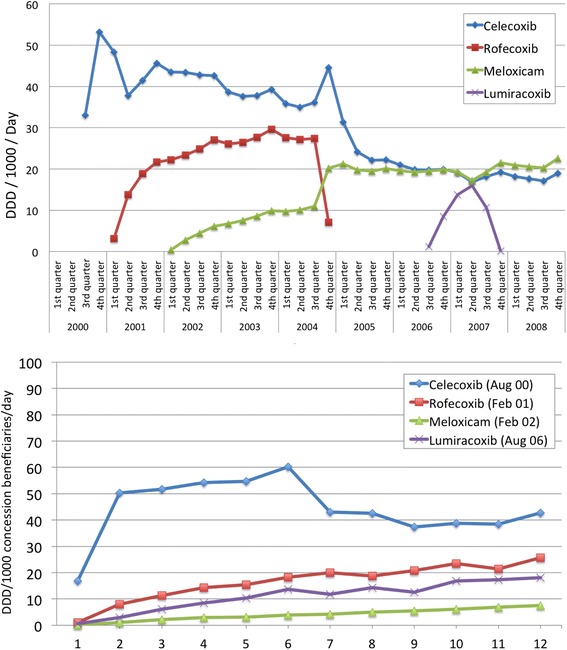
Utilisation of the COX-2 inhibitors (**a**) in the Australian concession beneficiary population from 2000 to 2008; and **b** over the initial year of each drug’s subsidy on the Australian Pharmaceutical Benefits Scheme (date of introduction shown)

Figure 1a) demonstrates the use of the COX-2 inhibitors in the Australian concession beneficiary population encompassing the time period when rofecoxib was withdrawn from the market. Celecoxib and rofecoxib were the market leaders, together accounting for over 50 % of the utilisation of NSAIDs in the years 2002–2004 [12]. When rofecoxib was withdrawn from the market celecoxib dispensing initially increased and then fell markedly in ensuing months. However, meloxicam, another COX-2 inhibitor showed an increased utilisation over the year following rofecoxib withdrawal. Lumiracoxib was subsidized in the second half of 2006 and withdrawn just over a year later, but reached utilisation similar to celecoxib and meloxicam in that short time.

Figure 1b) details the dispensing patterns in the concession population for each COX-2 inhibitor over the initial year of each drug’s availability subsidised on the PBS. Lumiracoxib was introduced and made available on the PBS in 2006, after rofecoxib had been withdrawn. Lumiracoxib showed a faster uptake rate than meloxicam, which was first subsidised by the PBS in 2002, before rofecoxib withdrawal.

**Fig. 2:**
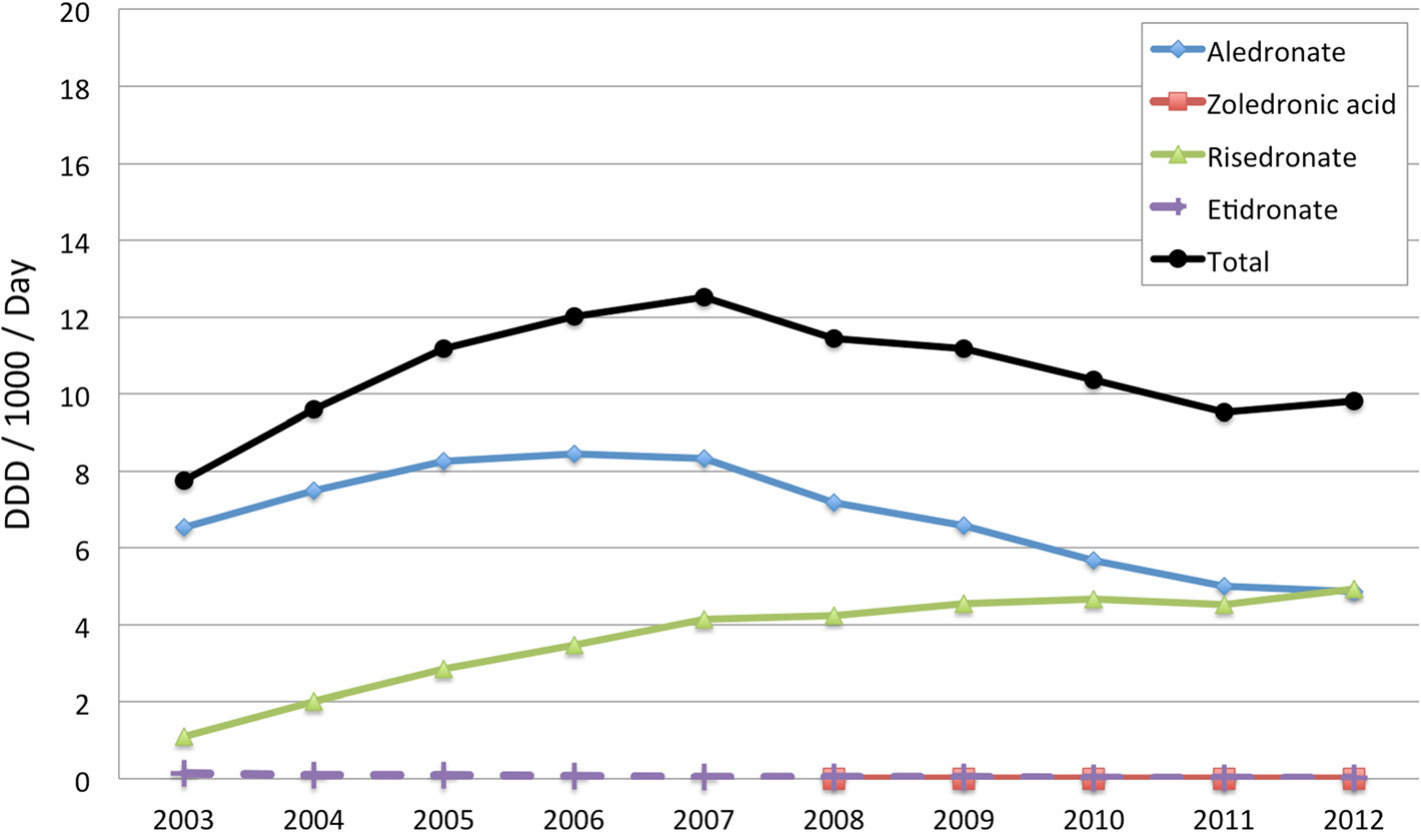
PBS subsidised utilisation of the bisphosphonates as anti-osteoporosis medications from 2003 to 2012

**Fig. 3:**
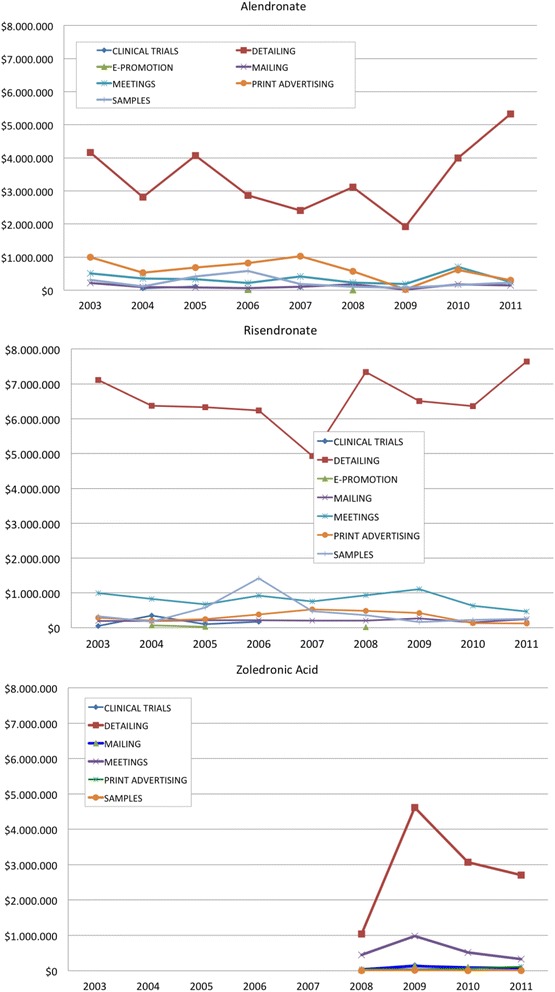
Australian marketing expenditures (AUD$) in different channels, by year, for bisphosphonates (data purchased from Cegedim^R^)

Figure 2 shows the PBS subsidised utilisation of the bisphosphonates as anti-osteoporosis medications (data for other subsidised indications, such as bone metastases, have not been included). Utilisation peaked in 2007 for total bisphosphonates. However, while alendronate use decreased after 2007, utilisation of risedronate increased and was still increasing at the end of the data analysis period. Zoledronic acid was first subsidised by the PBS in 2008 and had low but gradually increasing usage by 2012. Marketing expenditures (Fig. 3), gathered by the commercial company Cegedim^R^, show that expenditure in Australia on marketing, particularly detailing, increased substantially after 2007 for risedronate, and after an initial drop in 2008 began to increase also for alendronate. This followed the time that warnings about jaw osteonecrosis (and the safety alert in December 2007) were published by the Australian regulatory authority.

## Discussion

When adverse events to a medication cause sufficient concern for it to be withdrawn from the market, it might be expected that other medications in the same class, especially those introduced after the withdrawal, would be prescribed with more caution. Similarly, flagging concern (issuing warnings) about adverse events to a class of medications by regulatory agencies might also be expected to lead to caution in future prescribing of that class. There should perhaps be a decrease in industry promotion of those affected products with a preferred focus on more promulgation of best evidence about how best to use the medication class or alternatives.

In the example of COX-2 inhibitors, after rofecoxib was withdrawn it has been reported that many people stopped their medication [15]. However, from the sharp increases in dispensing of other COX-2 inhibitors recorded in this study (Fig. 1a), it seems that many rofecoxib users switched to other medications in the same class (unless these were all new users, but this is highly unlikely). Previous Australian data have shown changes in other NSAID utilisation following rofecoxib withdrawal [12, 20]. Similar switching after rofecoxib withdrawal has been reported in other studies [14], including studies on Danish use [21] and a comparison of six European countries [22]. However, evaluation of uptake of newer COX-2 inhibitors has not specifically been investigated previously.

As demonstrated by Fig. 1b, even though the uptake of prescribing of lumiracoxib in Australia was lower than the uptake of celecoxib and rofecoxib when they were first subsidised, the uptake rate was faster than that of meloxicam when it was first subsidised in 2002. Given that lumiracoxib was introduced after rofecoxib withdrawal in Australia, it might have been expected that prescribing would have proceeded with more caution. The increased use of meloxicam after rofecoxib withdrawal, shown in Fig. 1a, should also be of concern. Prescriber characteristics for new medications, including COX-2 inhibitors, in the Netherlands have been previously described, with a small subset of prescribers responsible for a high volume of initiations of new treatments [23]. These PBS data do not identify the prescriber, so it cannot be ascertained whether a smaller sub-group of prescribers accounted for most lumiracoxib and meloxicam prescribing in the wake of rofecoxib withdrawal. Ad hoc PBS data including some prescriber attributes may be purchased upon request, and this sub-group of data could be useful for further investigating this question. Rofecoxib was withdrawn due to safety issues and although there is still debate about whether this is a class effect, the withdrawal should have sounded a cautionary note about the widespread prescribing of COX-2 inhibitors [24–27]. Lumiracoxib itself was marketed in Australia for only 12 months before its withdrawal (Aug 2007) due to concerns about possible liver failure. Following lumiracoxib withdrawal, utilisation of celecoxib and meloxicam did not significantly decrease. This lack of response in celecoxib and meloxicam dispensing after a second withdrawal from the same drug class in a 2 year time period is also of concern.

In the second example used in this study, and as described previously, following negative publicity relating osteonecrosis of the jaw to bisphosphonate use and in particular alendronate use, a drop in alendronate dispensing was observed [10, 28]. This drop in alendronate dispensing occurred despite expansion of Australian publicly subsidised indications for bisphosphonates in 2008 (previously indicated for the treatment of established osteoporosis in people with fracture due to minimal trauma and then extended to the treatment of osteoporosis without fracture, in people aged 70 years or older who have a bone mineral density (BMD) T-score of −3.0 or less), and coincided with the dramatic rise in expenditure on detailing (Fig. 3), followed by a rise in utilisation of risedronate. This could suggest that adverse publicity had triggered a marketing response, especially for risedronate. The number of voluntary reports relating to osteonecrosis of the jaw (106) included oral alendronate (19) and oral risedronate (2) as implicated bisphosphonates, with the remainder being the IV formulations [8]. The larger numbers of reports with alendronate is probably due to the prevalence of use of alendronate and its more mature stage in the product life cycle, with more people exposed compared to risedronate. It would be a concern if this difference in voluntary adverse reaction reports was used to suggest any advantage of risedronate over the alternate bisphosphonates. Use of other classes of anti-osteoporosis medications has been investigated previously in a longitudinal population study of ageing women in Australia, and this did not show high use of these other classes at this time [10]. Some classes only received PBS subsidy late in the time period of interest (eg. 2009 teriparatide; 2007 strontium ranelate). Other adverse effects of bisphosphonates (eg. atypical fractures, oesophageal cancer) could have influenced prescribing but these did not receive the attention and publicity that osteonecrosis of the jaw did.

Zoledronic acid received PBS subsidy for osteoporosis indications in 2008, as a once yearly parenteral formulation; this was after the major publicity about osteonecrosis of the jaw. There was an initial spike in marketing expenditure, especially for detailing, as expected for new products. Use was substantially less than the oral bisphosphonates, however use was measured in DDD (4 mg) which may not be optimal for zoledronic acid. We have used DDD analyses to allow international comparisons to our data. However, a sensitivity analysis indicated that even if a ‘usual dose’ for zolendronic acid of 5 mg per year were used in calculations this would not account for the decline in total bisphosphonate usage (achieving 1.4 ‘usual daily doses’/1000 population/year by 2012; total bisphosphonate ‘use’ 11, rather than 10).

Media publicity about adverse effects can be useful in raising public awareness of potential medication safety issues, but the media have also been evaluated as not always reporting evidence-based decision making [29]. Osteonecrosis of the jaw is a serious adverse effect of bisphosphonates, but with a very low prevalence and only in a very specific subgroup [30, 31]. The problem with the publicity was that rational explanation of the risk was lost in the media coverage and that women with a high fracture risk, but very low risk of necrosis, perhaps ceased alendronate [30, 31].

The pharmaceutical industry is known to influence prescribing decisions [4], and their mandate is to act in the best interests of their shareholders. Innovation in pharmaceuticals is required but the cost of this and the effectiveness of current funding models (sales of existing drugs) have been questioned [32]. The amount spent on marketing can be immense and in some cases perhaps inappropriate [4, 33]. In the case of bisphosphonates, marketing expenditure was increased substantially for risedronate after publicity about osteonecrosis. Risedronate is now the market leader.

Regulatory agencies do have a role in preventing access to medications likely to have safety issues. A recent study identified that 26 % of drugs failing first cycle review for approval in the US did so for safety reasons, and a further 27 % failed due to safety and efficacy deficiencies [34]. However, medications with rare adverse events are not picked up by the usual clinical trials required for drug registration, and hence there is an urgent need for new ways of monitoring medications after market approval.

Concepts of risk and benefit need to be clearly defined and made transparent for consumers [2]. No medication is without risk, but the lessons learned from previous experience and new methods for post-market surveillance including updating of risks and benefits from new medications which evolve from utilisation in large numbers of ‘real life’ people need to be incorporated in a systematic and unbiased information resource [2, 4, 35]. In this paper, two different methods for tracking and evaluating use after adverse event reports have been used. The questions answered for the two examples were distinctly different and not designed to be addressed in the same way by the same data. For the bisphosphonates, expenditure on marketing could be an important motivator to prescribe (after the publicity about the adverse event) as all bisphosphonate products were still available but competing for market share and to grow the market. For the COX-2 inhibitors, one product, rofecoxib, was completely withdrawn. Therefore the question was not how the companies spent money to promote their products but rather how these products, as members of the same class, were being prescribed after that withdrawal. The two examples show that different data may be needed to explore and track what is happening after key adverse events, to ensure that subsequent interventions can successfully target better use of the medication in the same classes. For COX-2 inhibitors, dispensing data for the period after rofecoxib withdrawal were evaluated, and dispensing during the first 12 months of PBS subsidy were contrasted. For the bisphosphonates, dispensing data for the relevant time period 2003–2011 and also marketing data were evaluated to assess any relationships to key dates. These examples use different techniques relevant to each specific medication group and other techniques and datasets may be more relevant for other drug(s).

## Conclusions

Tracking of medication classes after adverse events have been reported for one member of the class is important and can give valuable information. This information could be used, if collected prospectively in real time, to indicate appropriate proactive interventions for other members of the class (such as education, or regulatory response like prior authorisation) to ensure best use of these targeted medications. One example, the use of COX-2 inhibitors after withdrawal of rofecoxib, has shown that, in Australia, use of another COX-2 inhibitor, meloxicam, increased and that the subsequent introduction of a new COX-2 inhibitor, lumiracoxib, led to widespread use of this medicine before it too was withdrawn because of adverse events. A more cautious approach Australia-wide could have been advocated if these trends had been prospectively monitored and interventions to assist prescribing and use developed at the time. The second example, the bisphosphonates and osteonecrosis of the jaw, has shown that Australian marketing expenditures by the pharmaceutical companies increased markedly at the time of the adverse publicity. A real time analysis of content, with perhaps opportunities for unbiased information around product class would have been of assistance for prescribers and consumers. There are Australian datasets available to help track and monitor medications classes which need to be used prospectively in ‘real time’ to ensure best quality use of medicines in the community.

## Additional file

Additional file 1:
**ATC codes and Pharmaceutical Benefits Scheme (PBS) item numbers for bisphosphonates.** (DOCX 13 kb) Additional file 2:
**ATC codes and Pharmaceutical Benefits Scheme (PBS) item numbers for COX-2 inhibitors.** (DOCX 13 kb)
