# Supplementary material for: Actions following adverse drug events – how do these influence uptake and utilisation of newer and/or similar medications?
Source: BMC Health Serv Res. 2015 Nov 6;15:498. doi: 10.1186/s12913-015-1165-9 (PMC4635584; doi:10.1186/s12913-015-1165-9)
Supplement: Additional file 1: — ATC codes and Pharmaceutical Benefits Scheme (PBS) item numbers for bisphosphonates. (DOCX 13 kb) [file 12913_2015_1165_MOESM1_ESM.docx]

Additional File One. ATC codes and Pharmaceutical Benefits Scheme (PBS) item numbers for bisphosphonates

| Generic name | ATC code | PBS item number | WHO ATC DDD (2014) |
| --- | --- | --- | --- |
| Alendronate | M05BA04 | 8511Y, 8102K | 10mg |
|  | M05BB03 | 9012H, 9183H, 2194L, 2224C |  |
|  | M05BB05 | 9351E, 2273P |  |
| Zoledronic acid | M05BA08 | 9288W | 4mg |
| Risedronate | M05BA07 | 4443W, 4444X, 2191H, 8481J, 8621R, 8972F, 9391G | 5mg |
|  | M05BB02 | 8899J, 8973G, 4059P, 2220W |  |
|  | M05BB04 | 9147K, 8974H, 4380M, 2254P |  |
| Disodium etidronate | M05BB01 | 8056B | 400mg |
