# Supplementary material for: Actions following adverse drug events – how do these influence uptake and utilisation of newer and/or similar medications?
Source: BMC Health Serv Res. 2015 Nov 6;15:498. doi: 10.1186/s12913-015-1165-9 (PMC4635584; doi:10.1186/s12913-015-1165-9)
Supplement: Additional file 2: — ATC codes and Pharmaceutical Benefits Scheme (PBS) item numbers for COX-2 inhibitors. (DOCX 13 kb) [file 12913_2015_1165_MOESM2_ESM.docx]

Additional File Two. ATC codes and Pharmaceutical Benefits Scheme (PBS) item numbers for COX-2 inhibitors

| Generic name | ATC code | PBS item number | WHO ATC DDD (2014) |
| --- | --- | --- | --- |
| Celecoxib | M01AH01 | 8439E (100mg, 60), 8440F (200mg 30) | 200mg |
| Rofecoxib | M01AH02 | 8471W (12.5mg, 30), 8472X (25mg 30), 8473Y (12.5mg/5mL, 150mL), 8474B (25mg/5mL, 150mL) | 25mg |
| Meloxicam | M01AC06 | 8561N (7.5mg, 30), 8562P (15mg, 30), 8887R (7.5mg, 30), 8888T (15mg, 30) | 15mg |
| Lumiracoxib | M01AH06 | 9032J (200mg, 30) | 100mg |
